# Gender Differences in Psychosocial Predictors of Sexual Activity and HIV Testing Among Youth in Kenya

**DOI:** 10.3389/frph.2021.636462

**Published:** 2021-05-11

**Authors:** Tiffany Chenneville, Hunter Drake

**Affiliations:** ^1^Department of Psychology, University of South Florida, St. Petersburg, FL, United States; ^2^Department of Community and Family Health, University of South Florida, Tampa, FL, United States

**Keywords:** HIV, youth, Kenya, sexual activity, HIV testing, gender

## Abstract

Sub-Saharan Africa (SSA) carries a disproportionate burden of HIV in the world relative to its population. Youth are at particular risk. Understanding HIV risk factors, as well as factors affecting HIV testing among SSA youth, is important given that HIV testing, linkage to care, and viral suppression are part of the global strategy to end HIV. Because young women face disparate sexual and reproductive health outcomes, exploring gender differences related to HIV risk, and testing is vital. Using existing program evaluation data from a larger project, the purpose of this study was to explore gender differences related to sexual activity and HIV testing among youth in SSA. Participant data from 581 youth ages 13–24 in Kenya was analyzed using descriptive statistics, analysis of covariance, and binomial logistic regression. Findings revealed that young men were more likely to report sexual activity than young women. Age was a predictor of sexual activity for all youth. However, among psychosocial variables, depression predicted sexual activity for young women while stress predicted sexual activity for young men. Although there were no gender differences in HIV testing after controlling for demographic and psychosocial variables, there were some differences between young women and young men with regard to predictors of HIV testing. Age and full-time self-employment predicted HIV testing among young women, while part-time self-employment, education, and substance abuse risk predicted HIV testing among young men. Findings suggest a need for gender and youth friendly strategies for addressing the HIV treatment cascade and care continuum.

## Introduction

Sub-Saharan Africa (SSA) carries over 70% of the HIV disease burden yet accounts for only 12% of the world population (1). Youth are at particular risk for HIV. As of 2017, there were an estimated 3.9 million youth ages 15–24 years living with HIV worldwide. Among the estimated 610,000 new cases of HIV among youth in this age range in 2016, approximately 84% were in SSA [United Nations Children's Fund, (2)]. Sexual activity, particularly early sexual debut, is a known risk factor for HIV. Based on survey data from a representative sample of nearly 7,700 sexually active South African youth, Pettifor et al. (3) found that early sexual debut was correlated with experiences of forced sex and sex with older partners, both risk factors for HIV infection. Pettifor et al. (3) also reported gender differences related to early sexual debut. Although more young men reported early sexual debut than young women, early sexual debut was elevated among young women who experienced forced sex (3). Early sexual debut was elevated for both young men and young women whose first sexual partner was older (3).

Recognizing the importance of HIV testing for ending the AIDS epidemic, the Joint United Nations Programme on HIV/AIDS (4) set a 90-90-90 treatment target for 2020 aimed at assuring that 90% of people living with HIV (PLWH) would be aware of their status (via HIV testing), 90% of PLWH would be receiving antiretroviral therapy, and 90% of PLWH would be virally suppressed. Although there has been progress toward these goals with increased HIV testing and linkage to care, the UNAIDS targets have not yet been met. As of July 2020, only 81% of PLWH were aware of their status, only 67% were on antiretroviral therapy, and only 59% were virally suppressed [Joint United Nations Programme on HIV/AIDS, (5)]. The failure to meet these targets speaks to the need to better understand factors related to HIV testing.

In an examination of factors related to behavioral intentions related to HIV testing among youth in Kenya, Nall et al. (6) found that HIV knowledge and substance use served as facilitators to HIV testing intentions while social support and depression served as barriers to HIV testing intentions. Although HIV stigma was independently correlated with the intention to test for HIV, it did not serve as a significant predictor based on findings from a regression analysis (6). Unfortunately, Nall et al. (6) did not examine gender differences related to HIV testing intentions. In a study of HIV testing among heterosexual youth in an urban city in the US, Decker et al. (7) found that young women (69.4%) were more likely to report HIV testing within the past year than young men (49.6%). Similarly, in a cross-sectional study of youth in SSA, young women were more likely to report HIV testing than young men (8).

Asaolu et al. (8) argued the need for further exploration into the contextual factors related to HIV testing in SSA, especially considering that the age of consent for sexual activity is lower than the age of consent for HIV testing among youth. In response to this and to address gaps in the existing literature on HIV risk among young people in the African context, the purpose of this study was to further explore gender differences related to sexual activity and HIV testing among youth in Kenya using existing program evaluation data from the HIV SEERs Project (9).

## Methods

### Study Design, Participants, and Setting

This study employed a cross-sectional design using existing program evaluation data from a larger project designed to assess the utility of a community-based HIV stigma reduction program in Nakuru, Kenya, which is located approximately 55 miles northwest of Nairobi and has a population of approximately 300,000 people (9, 10). Data for the larger project were gathered from 1,526 people recruited by trained local facilitators from schools and community centers to participate in the SEERs Project (Stigma-reduction through Education, Empowerment, and Research). Participation was completely voluntary. HIV status was not part of inclusion or exclusion criteria. Most participants reported being HIV negative. Program participants ranged in age from 12 to 36 years with an average age of 17 years.

For the current study, data from all participants aged 13–24 were extracted from the larger dataset. The University of South Florida Institutional Review Board reviewed the original project and determined it exempt given its use of existing anonymous program evaluation data.

### Procedures and Measures

As part of the larger project [see (10)], participants completed a demographic questionnaire and pre, post, and 3-month follow up measures. All measures were translated into Swahili using a back translation method and made available to participants; however, all participants chose to complete the measures that were printed in English, which is likely related to the fact that English is the language of instruction in Kenya (11).

The current study examined pre-test data only. Specifically, this study involved an analysis of demographic and pre-test data from the following measures: Brief HIV Knowledge Questionnaire [HIV KQ-18; (12)], the AIDS-Related Stigma Scale [ARSS; (13)], the Social Provision Scale [SPS; (14, 15)], the Depression, Anxiety, and Stress Scale [DASS; (16)], subjective well-being [SWB; (17)], and the CRAFFT (18). For information about the reliability and validity of these measures, including their utility with the sample from the larger project, please see Chenneville et al. (10) and Nall et al. (6).

### Data Analysis and Interpretation

Analyses were conducted using SPSS version 26. Descriptive statistics were used to describe the sample and performance on the various measures. Independent samples *t*-tests were used to examine gender differences in psychosocial variables, including HIV knowledge, projected stigma, social support, depression, anxiety, stress, and substance use. Analysis of covariance was used to determine the effect of gender on sexual activity or having been tested for HIV controlling for demographic and psychosocial variables. Finally, binomial logistic regression was used to determine the predictive effect of covariates by gender. All alpha values were set at 0.05.

On the HIV KQ-18, low scores indicate low knowledge and high scores indicate high knowledge. Similarly, low scores indicate low levels of stigma (ARSS); social support (SPS); depression, anxiety, and stress (DASS); and substance use (CRAFFT) while high scores indicate high levels. On the SWB measure, low scores typically indicate high social support; however, items were transformed in the SPSS scoring script so that low scores indicate low levels of social support. This was done to allow for ease of interpretation (i.e., for all scores reported below, low scores indicate low levels of knowledge or mental health indicators and high scores indicate high levels).

## Results

### Participant Demographics

Participants (*N* = 581) were predominantly female (55.7%) and aged 13–24 years old (M = 16.99 ± 2.98)^1^ (see Table 1). For demographic items, participants had the option of not responding, which accounts for the variation in sample size numbers for different demographic variables. The large majority of respondents did not identify as gay or lesbian, and there were no gender differences on the endorsement of a sexual identity other than heterosexual. The religious makeup of the sample was predominantly Christian (95.32%). Few participants reported having a physical disability (1.88%) or a history of mental illness (0.96%). Most respondents were unemployed (72.58%), with significantly more females (77.57%) reporting being unemployed than males (66.52%). Of those who reported being employed, males (13.39%) were more likely to report being self-employed full-time than females (9.68%). The majority of respondents reported a primary (49.44%) or secondary (26.32%) school education with no gender differences between these groups.

**Table 1:** Demographics.

		**Females**		**Males**		**Total**	
		***N***	**(%)**	***N***	**(%)**	***N***	**(%)**
		324	(55.77)	257	(44.23)	581	(100.0)
Religion	Christianity	308	(95.65)	242	(94.90)	550	(95.32)
	Islamic	6	(1.86)	5	(1.96)	11	(1.91)
	Hinduism	3	(0.93)	0	(0.00)	3	(0.52)
	Buddhism	3	(0.93)	1	(0.39)	4	(0.69)
	Other	0	(0.00)	2	(0.78)	2	(0.35)
	None	2	(0.62)	5	(1.96)	7	(1.21)
	Total	322	(100.00)	255	(100.00)	577	(100.00)
Education	No formal education	24	(8.36)	24	(9.80)	48	(9.02)
	Primary school	123	(42.86)	140	(57.14)	263	(49.44)
	Secondary school	90	(31.36)	50	(20.41)	140	(26.32)
	Technical college\university	50	(17.42)	31	(12.65)	81	(15.22)
	Total	287	(100.00)	245	(100.00)	532	(100.00)
Employment	Full-time employee	14	(5.15)	13	(5.80)	27	(5.44)
	Full-time self-employed	18	(6.62)	30	(13.39)	48	(9.68)
	Part-time employee	12	(4.41)	14	(6.25)	26	(5.24)
	Part-time self-employed	17	(6.25)	18	(8.04)	35	(7.06)
	Unemployed	211	(77.57)	149	(66.52)	360	(72.58)
	Total	272	(100.00)	224	(100.00)	496	(100.00)
Physical disability		4	(1.39)	6	(2.46)	10	(1.88)
Mental illness		3	(1.05)	2	(0.84)	5	(0.96)
Gay or lesbian		9	(2.90)	6	(2.44)	15	(2.70)

### Gender Differences on Measures

Independent samples *t*-tests were conducted to explore gender differences on HIV knowledge (HIV KQ-18), projected stigma (ARSS), social support (SPS), depression (DASS), anxiety (DASS), stress (DASS), happiness (SWB Scale), and substance use (CRAFFT). Females (*M* = 13.90 ± 2.60) scored significantly higher than males (*M* = 13.31 ± 2.83) on HIV knowledge (see Table 2). Females (*M* = 33.49 ± 6.83) scored significantly lower than males (*M* = 34.87 ± 7.59) on social support. Females also scored significantly lower on all DASS subscales (depression: *M* = 6.54 ± 9.28; anxiety: *M* = 5.81 ± 8.11; stress: *M* = 6.99 ± 9.24) than males (depression: *M* = 9.10 ± 9.77; anxiety: *M* = 9.73 ± 10.24; stress: *M* = 9.97 ± 9.70). On the CRAFFT, females (*M* = 0.25 ± 0.62) scored significantly higher than males (*M* = 0.43 ± 0.75) on the 12-month substance use history items of the CRAFFT (Part A). However, there were no significant differences by gender on the CRAFFT (Part B), which measures substance use risk. Likewise, there were no significant gender differences in measures of projected stigma and happiness (Table 2 contains additional statistics).

**Table 2:** Gender differences on psychosocial variables.

**Measure**	**Gender**	***N***	***M***	***SD***	***t***	***df***	***p***	***M_***diff***_***	**95% CI of** ***M**_***diff***_*	
									**Lower**	**Upper**
HIV Knowledge (HIV KQ-18)	Female	324	13.90	2.60	2.63	579.00	0.009^**^	0.594	0.150	1.038
	Male	257	13.31	2.83						
Projected stigma (AIDS-related stigma scale)	Female	291	1.23	1.24	−1.06	519.00	0.288	−0.112	−0.320	0.095
	Male	230	1.34	1.14						
Social support (social provision scale)	Female	318	33.49	6.83	−2.27	568.00	0.023^*^	−1.375	−2.564	−0.186
	Male	252	34.87	7.59						
Depression (DASS)	Female	310	6.54	9.28	−3.14	549.00	0.002^**^	−2.560	−4.162	−0.958
	Male	241	9.10	9.77						
Anxiety (DASS)	Female	311	5.81	8.11	−4.87	450.07	0.000^***^	−3.914	−5.492	−2.335
	Male	242	9.73	10.24						
Stress (DASS)	Female	310	6.99	9.24	−3.68	551.00	0.000^***^	−2.974	−4.563	−1.384
	Male	243	9.97	9.70						
Happiness	Female	265	7.80	2.48	−0.50	468.00	0.620	−0.112	−0.554	0.330
(Subjective well-being scale)	Male	205	7.91	2.34						
Substance use (CRAFFT part A)	Female	308	0.25	0.62	−3.06	450.51	0.002^**^	−0.185	−0.305	−0.066
	Male	236	0.43	0.75						
Substance use (CRAFFT part B)	Female	296	1.44	0.84	−1.12	525.00	0.263	−0.089	−0.245	0.067
	Male	231	1.53	0.98						

* *Significant at the 0.05 level.*

** *Significant at the 0.01 level.*

*** *Significant at the 0.001 level*.

### Gender and Sexual Activity

After dummy coding categorical demographic variables, an ANCOVA was conducted to determine the effect of gender (Female = 0, Male = 1) on being sexually active (No = 0, Yes = 1) after controlling for age, religion, physical disability, mental disability, education, employment, HIV knowledge, projected stigma, social supports, depression, anxiety, stress, SWB, and substance use (see Table 3). Controlling for covariates, significantly fewer females (*M* = 0.215) reported being sexually active than males (*M* = 0.403). Binomial logistic regressions were conducted to examine each covariate as a potential significant predictor of being sexually active by gender after controlling for age (see Table 4). For females, age and the depression subscale of the DASS were the only significant predictors of being sexually active. For every year increase in age, female participants were 1.501 times more likely to report being sexually active (*OR* = 1.501, *p* < 0.001, 95% CI [1.333, 1.689]). To a lesser degree, depression also predicted being sexually active in females. Controlling for age, with every point increase on the DASS depression subscale, females were 1.046 times more likely to report being sexually active (*OR* = 1.046, *p* = 0.013, 95% CI [1.010, 1.084]). For males, age and the stress subscale of the DASS were significant predictors of being sexually active. For every year increase in age, male participants were 1.480 times more likely to report being sexually active (*OR* = 1.480, *p* < 0.001, 95% CI [1.309, 1.675]) (see Table 4). To a lesser degree, stress also predicted being sexually active in males. Controlling for age, with every point increase on the DASS stress subscale, males were 1.038 times more likely to report being sexually active (*OR* = 1.038, *p* = 0.026, 95% CI [1.005, 1.073]).

**Table 3:** ANCOVA: gender differences on sexual activity and having been tested for HIV.

			**95% CI**				**95% CI** ***M**_***diff***_*^**a**^				
**Gender**	***M***	***SE***	**Lower bound**	**Upper bound**	***M_***diff***_***	***SE***	**Lower bound**	**Upper bound**	***F***	***p***	**partial eta-squared**
**Sexual activity**											
Female	0.215	0.033	0.150	0.279	−0.188^*^	0.052	−0.291	−0.086	13.192	0.000^*^	0.050
Male	0.403	0.037	0.331	0.475	0.188^*^	0.052	0.086	0.291			
**Having been tested for HIV**											
Female	0.579	0.036	0.507	0.650	0.033	0.058	−0.081	0.147	0.321	0.572	0.001
Male	0.546	0.041	0.464	0.627	−0.033	0.058	−0.147	0.081			

a *Adjustment for multiple comparisons: Bonferroni*.

* *Significant at the 0.05 level*.

**Table 4:** Predictors of sexual activity and having been tested for HIV.

**Predictor**		***B***	***S.E*.**	***p***	***OR***	**Lower**	**Upper**
**Sexual activity among females** ^**a**^							
Age	0.406	0.060	0.000^***^	1.501	1.333	1.689	
Sexual identity		−0.099	0.886	0.911	0.905	0.159	5.143
Mental illness		2.401	1.449	0.098	11.032	0.644	188.967
Education	Primary school	−1.219	0.895	0.173	0.295	0.051	1.707
	Secondary School	0.330	0.885	0.709	1.391	0.245	7.887
	Technical college/university	1.102	0.980	0.261	3.010	0.441	20.556
Employment	Employed full-time	−0.208	0.889	0.815	0.812	0.142	4.633
	Employed part-time	0.228	0.723	0.752	1.257	0.304	5.187
	Self-employed full-time	−0.432	0.750	0.564	0.649	0.149	2.822
	Self-employed part-time	0.899	0.706	0.203	2.456	0.615	9.807
HIV knowledge		0.000	0.068	0.997	1.000	0.876	1.141
Projected stigma		−0.042	0.155	0.788	0.959	0.708	1.299
Social support		−0.023	0.026	0.376	0.978	0.930	1.028
Subjective well-being		0.058	0.291	0.843	1.059	0.598	1.875
Depression		0.045	0.018	0.013^*^	1.046	1.010	1.084
Anxiety		0.025	0.021	0.232	1.026	0.984	1.070
Stress		0.024	0.019	0.193	1.025	0.988	1.063
Substance use		0.192	0.201	0.339	1.212	0.817	1.797
**Sexual activity among males** ^**b**^							
Age	0.392	0.063	0.000^***^	1.480	1.309	1.675	
Sexual identity		1.592	0.979	0.104	4.916	0.722	33.478
Physical disability		1.259	0.999	0.208	3.522	0.497	24.963
Education	Primary school	0.044	0.610	0.942	1.045	0.316	3.452
	Secondary school	0.821	0.712	0.249	2.272	0.563	9.165
	Technical college/university	0.516	0.808	0.523	1.675	0.344	8.165
Employment	Employed full-time	−0.278	0.720	0.700	0.758	0.185	3.105
	Employed part-time	0.073	0.698	0.916	1.076	0.274	4.227
	Self-employed full-time	0.336	0.467	0.472	1.400	0.560	3.500
	Self-employed part-time	0.727	0.639	0.255	2.069	0.591	7.243
HIV knowledge		−0.019	0.059	0.754	0.982	0.874	1.102
Projected stigma		−0.046	0.151	0.760	0.955	0.710	1.284
Social support		0.002	0.021	0.930	1.002	0.962	1.044
Subjective well-being		0.214	0.317	0.500	1.239	0.665	2.307
Depression		0.024	0.016	0.140	1.024	0.992	1.057
Anxiety		0.014	0.015	0.372	1.014	0.984	1.045
Stress		0.037	0.017	0.026^*^	1.038	1.005	1.073
Substance use		0.246	0.158	0.118	1.279	0.939	1.742
**Females having been tested for HIV** ^**c**^							
Age	0.340	0.050	0.000^***^	1.406	1.274	1.551	
Sexual identity		−0.007	0.778	0.993	0.993	0.216	4.561
Physical disability		−0.097	1.154	0.933	0.908	0.094	8.723
Education	Primary school	−0.305	0.490	0.533	0.737	0.282	1.926
	Secondary school	−0.057	0.599	0.925	0.945	0.292	3.057
	Technical college/university	0.057	0.729	0.938	1.058	0.253	4.418
Employment	Employed full-time	0.620	0.657	0.345	1.860	0.513	6.745
	Employed part-time	0.534	0.861	0.535	1.705	0.316	9.212
	Self-employed full-time	2.637	1.068	0.014^*^	13.975	1.723	113.319
	Self-employed part-time	−0.038	0.616	0.950	0.962	0.288	3.217
HIV knowledge		0.020	0.050	0.691	1.020	0.924	1.126
Projected stigma		0.031	0.107	0.772	1.032	0.836	1.272
Social support		−0.003	0.019	0.851	0.997	0.961	1.033
Subjective well-being		0.201	0.221	0.363	1.222	0.793	1.883
Depression		0.015	0.014	0.272	1.015	0.988	1.043
Anxiety		0.023	0.016	0.152	1.023	0.992	1.056
Stress		0.016	0.014	0.243	1.016	0.989	1.044
Substance use		0.072	0.164	0.660	1.075	0.780	1.482
**Males having been tested for HIV** ^**d**^							
Age	0.179	0.048	0.000^***^	1.197	1.089	1.315	
Sexual identity		1.622	1.120	0.148	5.063	0.564	45.465
Physical disability		0.596	0.893	0.504	1.815	0.315	10.453
Mental illness		0.328	1.427	0.818	1.388	0.085	22.737
Education	Primary school	0.362	0.475	0.446	1.436	0.566	3.640
	Secondary school	1.187	0.595	0.046	3.278	1.020	10.527
	Technical college/university	1.582	0.719	0.028	4.863	1.189	19.887
Employment	Employed full-time	0.958	0.629	0.128	2.605	0.760	8.930
	Employed part-time	0.303	0.575	0.598	1.354	0.439	4.179
	Self-employed full-time	−0.122	0.418	0.771	0.885	0.391	2.008
	Self-employed part-time	2.543	1.055	0.016^*^	12.714	1.607	100.593
HIV knowledge		0.080	0.050	0.109	1.084	0.982	1.196
Projected stigma		0.126	0.127	0.319	1.135	0.885	1.455
Social support		−0.013	0.018	0.477	0.987	0.954	1.022
Subjective well-being		−0.247	0.268	0.357	0.781	0.462	1.322
Depression		0.014	0.014	0.300	1.015	0.987	1.043
Anxiety		0.023	0.013	0.090	1.023	0.996	1.050
Stress		0.014	0.014	0.305	1.014	0.987	1.042
Substance use		0.422	0.165	0.010^**^	1.525	1.105	2.106

a *All results for age are when age is the only predictor. All other regression models reported are controlled for age. Models for coefficients physical disability and religion could not be determined due to quasi-complete separation of the data.*

b *Models for mental illness and religion could not be determined due to quasi-complete separation of the data.*

c *Models for mental illness and religion could not be determined due to quasi-complete separation of the data.*

d *The model for religion could not be determined due to quasi-complete separation of the data.*

* *Significant at the 0.05 level.*

** *Significant at the 0.01 level.*

*** *Significant at the 0.001 level*.

### Gender and HIV Testing

After dummy coding categorical demographic variables, an ANCOVA was conducted to determine the effect of gender (Female = 0, Male = 1) on ever having been tested for HIV (No = 0, Yes = 1) after controlling for age, religion, physical disability, mental disability, education, employment, HIV knowledge, projected stigma, social supports, depression, anxiety, stress, SWB, and substance use (see Table 3). Controlling for covariates, there was not a statistically significant difference in having been tested for HIV between females (*M* = 0.579, *Mdiff* = 0.033, 95% CI [−0.081, 0.147], *p* = 0.572) and males (*M* = 0.546, *Mdiff* = −0.033, 95% CI [−0.147, 0.081], *p* = 0.572). Binomial logistic regressions were conducted to examine each covariate as a potential significant predictor of HIV testing by gender controlling for age (see Table 4). For females, age (*N* = 315; *M* = 17.10 ± 3.044, *Range*: 13–24) and being self-employed full-time were the only significant predictors of having been tested for HIV. For every year increase in age, female participants were 1.406 times more likely to have been tested for HIV (*OR* = 1.406, *p* < 0.001, 95% CI [1.274, 1.551]). Controlling for age, only being self-employed full-time remained a significant predictor of having been tested for HIV. Females who were self-employed full-time were 13.975 times more likely to have been tested than those who were unemployed (*OR* = 13.975, *p* = 0.014, 95% CI [1.723, 113.319]). Neither working full-time as an employee nor working part-time (as an employee or self-employed) predicted any significant difference in testing for females. For males, age (*N* = 248; *M* = 16.82 ± 2.876, *Range*: 13–24), level of education (*N* = 245), employment (*N* = 218), and substance use as identified by CRAFFT Part B scores (*N* = 231; *M* = 1.53 ± 0.982, *Range*: 0–6) were significant predictors of having been tested for HIV (see Table 4). For every year increase in age, male participants were 1.197 times more likely to have been tested for HIV (*OR* = 1.197, *p* < 0.001, 95% CI [1.089, 1.315]). Males who completed secondary school were 3.278 times more likely to have been tested than those without formal education (*OR* = 3.278, *p* = 0.046, 95% CI [1.020, 10.527]). Males who attended technical college or university were 4.863 times more likely to have been tested for HIV than those without formal education (*OR* = 4.863, *p* = 0.028, 95% CI [1.189, 19.887]). For employment, only those who were self-employed part-time were significantly different from those who were unemployed. Males who were self-employed part-time were 12.714 times more likely to have been tested than unemployed males (*OR* = 12.714, *p* = 0.016, 95% CI [1.607, 100.593]). Finally, for every point increase on the CRAFFT Part B as a measure of substance use, males were 1.525 times more likely to have been tested for HIV (*OR* = 1.525, *p* = 0.010, 95% CI [1.105, 2.106]).

## Discussion

This study aimed to explore gender differences related to sexual activity and HIV testing among youth ages 13–24 in Kenya by examining existing program evaluation data from a larger project [see (9)]. Consistent with Harrison et al.'s (19) study of the impact of gender (among other factors) on HIV risk among South African youth, results from the current study revealed gender differences in reports of sexual activity. Specifically, young men were more likely to report a history of sexual activity than young women. Age predicted sexual activity for both young men and women, which is not surprising given data that suggests sexual activity increases significantly as young people age through their adolescent years (20). Stress was a significant predictor of sexual activity among young men, while depression was a significant predictor of sexual activity for women. The latter builds upon Foley et al.'s (21) finding that depressive symptoms served as a longitudinal predictor of risky behaviors among a sample of sexually active African American adolescents. In Foley et al.'s (21) study, depressive symptoms predicted sex with multiple partners for female adolescents only. However, depressive symptoms had an indirect effect on condomless sex for both female and male adolescents (21). Although not focused on gender differences, Blignaut et al. (22) found that indicators of depression and suicidal ideation increased the likelihood of being sexually active among incoming first-year students at a South African university. However, there is some evidence to suggest that sexual activity predicts depression among youth, not the other way around (23). It is also possible that the relationship is bidirectional.

In this study, there were no significant differences in reports of past HIV testing based on gender after controlling for demographic and psychosocial variables. However, there were differences in the predictors of HIV testing based on gender. For females, age and full-time self-employment predicted HIV testing. Age as a predictor of HIV testing is not surprising given the age of consent laws for HIV testing (8) and the fact that youth are more likely to be sexually active with increasing age (20). The fact that full-time self-employment only (as opposed to working full-time as an employee or working part-time as an employee or self-employed) predicted HIV testing for young women is interesting and difficult to explain. Although self-employment may afford more flexibility with regard to time for HIV testing, the fact that part-time self-employment did not predict HIV testing suggests that time may not be the most important factor.

For males, education, employment, and substance use risk predicted HIV testing. Given that information about HIV is likely to be part of sexuality education curricula in formal school settings, the finding that education predicted HIV testing is consistent with studies showing HIV knowledge to be a significant predictor of HIV testing [e.g., (24, 25)] or behavioral intentions related to HIV testing (6). Although the likelihood of having been tested increases significantly with increasing levels of education, time and exposure to opportunities to be tested must also be taken into account. However, this does not discount the importance of education and its relationship to HIV testing. Whereas, full-time self-employment predicted HIV testing for young women, part-time self-employment predicted HIV testing for young men. Again, this is an interesting finding and difficult to explain. Regarding substance use as a predictor of HIV testing in this study, Luseno and Wechsberg (26) also reported an association between substance use and HIV testing among South African women. In addition, Nall et al. (6) found that substance use was a significant predictor, but for behavioral intentions related to HIV testing as opposed to a history of HIV testing. Unlike these findings, Altice et al. (27) found that HIV testing was less likely among people who used substances. Only Nall et al.'s (6) study focused on youth. The well-established link between substance use and HIV risk behaviors within and outside the sexual context (28) probably explains the impact of substance use on HIV testing in this study.

There are several limitations to this study. First, this study relied on self-report data from a larger project. Although data collected was anonymously, it is possible that youth were reluctant to respond honestly to questions about sexual activity and HIV testing due to real or perceived social (and gendered) norms about sexual activity outside of marriage and fear of HIV-related stigma. Second, available data for this study did not include information about protective factors (e.g., condom use, pre-exposure prophylaxis).

Despite limitations, current findings contribute to the existing literature on gender differences related to HIV risk and HIV testing, which are important factors within the framework of the HIV treatment cascade and care continuum (29). Although more research is needed in this area, results from this study support the need for novel and gender-based approaches that take into account age for HIV prevention and control, specifically with regard to addressing HIV risk factors and HIV testing. For example, programs designed to address sexual activity among youth may want to take into account the differential impact of mental health symptoms between young men (i.e., stress) and young women (i.e., depression). Further, programs designed to increase HIV testing also should consider methods that are gender-friendly and age appropriate. For example, Hensen et al. (30) found that mobile-based HIV testing and home-based strategies, in addition to offering HIV testing at health facilities, were effective for increasing HIV testing among men in SSA. Research is needed to determine if similar strategies effectively improve the uptake of HIV testing among women and, particularly, among young women. In closing, the exploration of gender differences related to HIV prevention and treatment should be considered within ongoing conversations about the importance of modifying African patriarchies to address the HIV epidemic in Africa (31).

## Data Availability Statement

The raw data supporting the conclusions of this article will be made available by the authors, without undue reservation.

## Ethics Statement

The University of South Florida Institutional Review Board reviewed the original project and determined it exempt given its use of existing anonymous program evaluation data.

## Author Contributions

TC is the Principal Investigator for the HIV SEERs Project from which program evaluation data, which served as the basis for this study, was drawn. TC and HD conceptualized the current study and contributed to the writing of this manuscript. HD was responsible for data analysis. Both authors contributed to the article and approved the submitted version.

## Conflict of Interest

The authors declare that the research was conducted in the absence of any commercial or financial relationships that could be construed as a potential conflict of interest.

## Footnotes

^1^M = Mean.
